# Neurocognitive Functioning in Schizophrenia and during the Early Phases of Psychosis: Targeting Cognitive Remediation Interventions

**DOI:** 10.1155/2013/819587

**Published:** 2013-09-05

**Authors:** Yulia Zaytseva, Natalya Korsakova, Mark Agius, Isaac Gurovich

**Affiliations:** ^1^Department of Outpatient Psychiatry and Organization of Psychiatric Care, Moscow Research Institute of Psychiatry, Poteshnaya Street 3, Moscow 107076, Russia; ^2^Lomonosov Moscow State University, Mokhovaya Street 11-9, Moscow 125009, Russia; ^3^Department of Psychiatry, Clare College, University of Cambridge and South Essex Partnership University Foundation Trust, Weller Wing Bedford Hospital, Kempston Road, Bedford MK42 9DJ, UK

## Abstract

Recent interest in the early course of schizophrenia accentuated altered cognition prior to the onset. Ultrahigh risk (UHR) individuals with attenuated positive symptoms and transient psychotic episodes demonstrate neurocognitive deficits across multiple domains such as memory, executive functioning, and processing speed which are consistent with similar disturbances identified in patients with a first episode of schizophrenia. Cognitive remediation (CR) approaches representing a broad set of activities are aimed to restore or improve cognitive functioning. CR proved to be effective in modulating the cognitive dysfunction in schizophrenia but is rarely used in ultrahigh risk individuals. From the clinical prospective, a better understanding of cognitive functioning in at-risk states is essential for the development of optimal early intervention models. In the review, we highlight the intervention targets, notably the specific cognitive deficits in at risk individuals which preceed the transition to psychosis and emphasize the need of the additional studies using CR approaches in UHR group aiming to enhance cognition and therefore mediate functional improvement.

## 1. Neurocognitive Deficits in Schizophrenia

Since the earliest description of schizophrenia, cognitive dysfunction has been noted as a core component of the illness [1]. At present, it is well established that individuals with schizophrenia demonstrate significant impairments across a broad range of cognitive domains, including memory, executive functions, attention, motor abilities, and spatial functions [2], in first episode patients [3, 4] and in their unaffected relatives [5, 6]. While the early studies reported “generalized” cognitive deficits in schizophrenia [7, 8], others underlined the “selectivity of dysfunction” in specific cognitive domains: memory [9, 10] and executive functioning [11, 12]. The overall impairment profile of individuals with schizophrenia supposedly represents the disruption of fundamental neural circuits encompassing cortical-cerebellar-thalamic-cortical subsystems [13]. Thus, many researchers proposed cognitive dysfunction as a potential endophenotype in schizophrenia due to their detection across schizophrenia spectrum groups [14, 15], high test-retest reliability [16, 17], and heritability [18].

In the last decade, there has been an increasing interest in cognitive disturbances in the early course of schizophrenia. While the neuropsychological deficits tend to be fairly stable in chronic schizophrenia [16, 19], it has been suggested that there might be more amenable changes during the prodromal period and shortly after the illness onset. A large retrospective study demonstrated impairment of cognition to be among the “first signs” in individuals who were later diagnosed with schizophrenia [20]. The presence of cognitive impairment prior to the onset of psychosis and early after its manifestation suggests that some neurocognitive abnormalities precede and are not solely a consequence of psychosis and thus may represent a trait marker for schizophrenia. Understanding the trajectory of cognitive changes in the development of schizophrenia may shed some light on the neurodevelopmental processes happening at the initial stages of the illness.

The staging model of schizophrenia defines the course of schizophrenia with regard to the clinical and functional level of deterioration and underlines the predictive validity of early and latter brain alterations [21–23]. Psychophysiological data largely drawn from neuroimaging studies differentiate between three major clinical stages which describe the brain volume changes over the course of the illness: ultrahigh risk stage (UHR) for psychosis or prepsychotic phase when people present with potentially prodromal symptoms, first episode or manifestation of psychosis and chronic schizophrenia. Specifically, the loss of grey matter in the prefrontal cortex, lateral temporal and medial temporal regions, superior temporal gyrus, and insula as well as the white matter abnormalities in the fronto-occipital fasciculus predicts the transition from ultrahigh risk to full-blown psychosis [24–27]. Consistent with the staging model, there has been an increasing body of evidence of persisting cognitive abnormalities in the early stages of schizophrenia: starting presumably in the prodromal phase, deteriorating and reaching the peak at the manifestation of the first psychotic episode, and remaining relatively stable at the course of schizophrenia [28]. However, the time course of the emergence of neurocognitive deficits is not well understood. Subtle cognitive deficits in working memory, attention, and processing speed are already detected in individuals with family risk for psychosis [29, 30] and children who developed adult schizophrenia [31]; these cognitive domains are predisposed to further deterioration [32]. Therefore, cognitive impairments in genetic high-risk subjects that are not confounded by psychosis or medications provide a strong support for a neurodevelopmental model of prepsychotic vulnerability for schizophrenia [32]. 

## 2. Cognitive Dysfunction in Ultrahigh Risk Individuals

The ultrahigh risk (UHR) status is characterised by subjectively experiencing disturbances in perception, thought processing, language, brief psychotic episodes that are distinct from classic psychotic symptoms, independent of abnormal thought content, and intact in reality testing and insight. UHR criteria include attenuated positive symptoms or brief limited intermittent psychotic symptoms and genetic risk [33]. The presence of at least one fully positive psychotic symptom several times per week for at least one month or at least one fully psychotic symptom for at least one day if this symptom is seriously disorganizing or dangerous constitutes the transition to psychosis criteria [33]. Individuals who meet the UHR criteria have a transition rate to psychosis of approximately 30–35% within a follow-up period of 1–3 years [34, 35]. 

Several studies have reported deficits in multiple cognitive domains in UHR samples, as compared to the general normal population, with the most pronounced impairments being of rapid information processing, including visual attention, processing speed, set shifting, and verbal fluency [32, 36, 37]. The most significant deficit among adolescents with attenuated positive symptoms was obtained in receptive language; these findings correspond with the study on childhood developmental deficits preceding schizophrenia [38] and suggest language processing as one of the earliest emerging alterations related to vulnerability to psychosis. Additionally, several authors observed spatial memory and visual-spatial processing deficits in UHR subjects compared to controls. Moreover, it has been shown that individuals who later develop a psychotic disorder performed more poorly on spatial working memory tests [39].

At a group level, the range of cognitive deficits in UHR subjects seems to have an intermediate position between healthy comparison subjects and patients with first episode of schizophrenia [15, 40]. Notably, Eastvold et al. [40] compared the baseline neurocognitive profiles of individuals at risk who later converted to psychosis (true prodromals) to those individuals who have experienced their first episode of psychosis and healthy comparison subjects. They showed that the true prodromals had the largest effect sizes versus the healthy comparison subjects in the verbal episodic memory scores and general intellectual functioning of the same magnitude as it was detected in the first episode sample. Jahshan et al. [41] also reported the significant time effects of executive functioning, processing speed, verbal learning, and general intelligence between the groups of UHR individuals, first episode patients, and normal controls. These results are in line with the other reports which stated verbal learning deficits at baseline assessment as well as the decline in verbal memory and general intellectual functioning in UHR subjects who later converted to psychosis. This cognitive decline was consistent with the worsening or the manifestation of the psychotic symptoms. However, impairments in attention seem not to have a predictive value in psychosis development [42–44].

Relatively fewer longitudinal studies on neuropsychological functioning in at-risk subjects have revealed an association between greater cognitive impairment at baseline and subsequent conversion to psychosis [45, 46], specifically, the decline in verbal abilities, memory, and intellectual functions [42, 43, 47, 48]. Niendam et al. [49] found that high-risk subjects improved over an 8-month period on measures of information processing speed, as well as visual and verbal learning/memory. However, the study of Hawkins et al. [50] in which participants were assessed at entry and at 6 and 12 months failed to prove a decline of the cognitive functioning during the period of transition to psychosis.

Regarding the stability of cognitive dysfunction in the groups of prodromals, first-episode patients, and controls, the results of the longitudinal study showed deterioration in working memory and processing speed in first-episode patients and UHR subjects who later converted to psychosis [41]. This is consistent with a cross-sectional investigation of neurocognitive functioning in patients classified as being in various stages of prodrome and postonset psychosis, which reported the increase of neurocognitive deficits at each “stage” of the illness [51]. Thus, cognitive functions do not follow a one-dimensional trajectory in schizophrenia but rather vary by cognitive domain and position in the course of the illness. 

 Not all studies have reported neurocognitive decline during the prodromal stage or simply a link between neurocognitive changes and conversion to psychosis [50, 52]. Some of these inconsistencies may be due to differences in the definition of the prodrome of schizophrenia. In studies where early and late prodromals were separated, significant difference in executive functioning with a higher percentage of perseverative and nonperseverative errors in Wisconsin Card Sorting Test (WCST) was obtained [53]. Frommann et al. [54] have recently reported that subjects in the late prodromal phase are impaired in all neurocognitive domains, whereas individuals in the early prodromal phase had a specific deficit only in processing speed/executive control.

Furthermore, these early changes in overall neurocognitive functioning seem to occur in synergy with changes in positive symptom severity in the UHR group. The substantial improvement in clinical symptoms and Global Assessment of Functioning Scale (GAF) scores over time in the UHR group highlights that the at-risk state can be transient in many young people; thus, cognitive changes may be difficult to detect [41]. In contrast with investigations showing a decline in IQ prior to the schizophrenia onset [55], a significant proportion of UHR subjects demonstrated an improvement in general intelligence over the 6-month followup.

Functional imaging studies in at-risk individuals while performing cognitive tasks also underline altered activations of brain areas which are prone to structural deficits [56]. Voxel-wise meta-analysis of fMRI studies in individuals at clinical high risk for psychosis revealed reduced blood oxygenation level-dependent response (BOLD) in the left inferior frontal gyrus and in a cluster spanning the bilateral medial frontal gyrus, bilateral superior frontal gyrus, and the left anterior cingulate [57].

Several systematic reviews and meta-analysis have demonstrated a relationship between standardized neurocognitive tests on attention, memory, and problem-solving as well as overall measures of cognition with the variety of parameters of social functioning and functional outcomes [58, 59]. It has also been shown that cognitive deficit could impede the acquisition of elementary social skills in schizophrenia [60, 61]. Moreover, neurocognitive impairment can negatively mediate social cognition and thereby exert a negative influence on functional outcomes [62]. In regard to the prodromal stage, clinical high-risk individuals display not only cognitive but also functional decline; notably, in the individuals with attenuated positive symptoms impaired processing speed is related to the difficulties in social and role functioning [63].

 In sum, cognitive deficits occur early in the prepsychotic phase of schizophrenia, mostly grasping memory, executive functioning, and general intellectual abilities although being less prominent as in the manifestation of psychosis. Receptive language and spatial memory functions seem to be prone to further deterioration which might have a predictive value in the transition to psychosis and hence require special focus in the preventive and treatment strategies. 

## 3. Cognitive Remediation and Its Effectiveness

Cognitive remediation (CR) represents a broad set of activities and exercises that aim to restore or improve cognitive functioning, that is, attention, working memory, planning, and executive functions by stimulating new learning and facilitating social functioning. The ultimate goal of the intervention though is a generalization of the obtained skills in the habitual community setting. To date, several models of cognitive remediation can be assessed. Some models emphasize training of isolated cognitive skills (verbal and visual working memory, executive function, attention, and processing speed), so-called drill practice using a number of trials of the same exercise to facilitate learning; other models offer cognitive training in conjunction with vocational training and social skills training [64]. The strategy-coaching approach uses cognitive-training exercises in a group setting and relies on development and maintenance of motivation in participants. 

The existing techniques of cognitive remediation combine cognitive and specific compensatory skills trainings which are addressed in accordance with the initial goal of the remediation therapy. Cognitive training, either computerized using CogRehab software [65], or therapist guided pen and pencil training includes tasks in the following cognitive domains: verbal and visual memory, language, visuo-motor skills, orientation, vigilance, processing speed, and so forth. The training is carried out by patients usually for 1-2 hours per day several times a week and integrated with weekly therapy sessions. The neuropsychological educational approach to rehabilitation (NEAR) method uses a strategy-coaching approach. After the computerized session of the training, participants elaborate and discuss in a small group the strategies that they have learned while practicing cognitive tasks and how these skills may be transposed to real life activities [66]. In neurocognitive enhancement therapy (NET) computerized cognitive remediation is used along with the vocational rehabilitation programs [67]. Cognitive enhancement therapy (CET) includes small-group sessions with a specific emphasis on social cognition and also comprises supportive therapy [68]. Pair cognitive remediation embodies cognitive training and a supported employment program aiming to facilitate social recovery in schizophrenia [69]. The number of sessions varies in different cognitive remediation approaches.

Recently, new programs exploiting cognitive remediation strategies have appeared. Thus, Cognitive adaptation training (CAT) is a home delivered cognitive rehabilitation strategy which is designed to train cognition in order to offer solutions to the daily life problems, specific and concrete to each individual [70]. Another intriguing approach is the brain fitness program (BFP), a drill practice method, used in order to improve brain plasticity. The aim of BFP is to restore and amplify auditory perception and working memory processing through six exercises of increasing complexity. It starts with the mastering of the formants in speech (such as phonemes, words, and sentences), continuing with the remembering of the sequence of verbal instructions, and ending with the processing of real world scenarios of conversational narratives [71]. 

 Several meta-analyses have been carried out to evaluate the effectiveness of cognitive remediation in schizophrenia [72, 73]. McGurk et al. [73] included 26 randomized control trials showing a medium range of the effect size of 6 from 7 estimated cognitive functions, specifically in attention, speed of processing, executive functioning, verbal working memory, verbal learning and memory, and visual learning and memory. The effects of cognitive remediation were similar across the 26 studies included in the analysis regardless of the duration of training and training methods, age of the participants, inpatient or outpatient provision of CR, and other complimentary rehabilitation programs. Follow-up data analysis of 6 studies also showed significant improvement of the global cognitive performance with the effect size 0.56 after initial treatment and 0.66 after 8 month followup [74]. It was also indicated that compared to drill and practice alone approaches, the strategies using coaching were less effective (effect size 0.48 versus 0.23) and longer programs were more beneficial for patients than shorter programs regardless of the type of the training (effect size 0.57 versus 0.29). In a recent study, Bowie et al. also indicated a favorable effect of combined treatment using cognitive remediation and functional skills training on cognitive functioning but not cognitive remediation alone in schizophrenia outpatients. Along with cognitive improvement, social competence increase was more durable with combined treatment during 12 months followup [75]. 

Taking into account cognitive heterogeneity in schizophrenia, it is crucial to identify whether particular groups of patients benefit from cognitive remediation therapy. The recent meta-analysis by Wykes et al. [76] aimed to demonstrate not only the effectiveness of CR but also to determine the way in which these programs help in different categories of patients, thus, indicating more malleable cognitive targets. Interestingly, cognitive impairment as an entry criterion did not affect cognition outcomes; larger effect sizes were detected in studies which included clinically stable patients with schizophrenia and schizoaffective disorder. Age seemed not to be a predictor and although greater numbers of symptoms were associated with smaller effect sizes, patients still benefitted from CR. Moreover, the results of the meta-analysis suggests that most effective programs combine CR with other rehabilitation modules. Thus, strategic approaches rather than drill practice favored better outcomes possibly due to the reciprocal boosting effects which contribute to transferring of the gained benefits into everyday life. It was also shown that cognitive remediation has a consistent effect on improving of functioning [73]. The impact of CR on functional outcomes was significantly greater when it was combined with psychosocial rehabilitation, therefore, providing a synergic effect [73, 76].

Nevertheless, the utilization of the computerized cognitive remediation training has not provided any evidence of the efficacy of the intervention in stable patients with schizophrenia [77, 78]. Although there has been a significant improvement of auditory processing speed, this effect did not lead to improved overall cognitive performance. One can hypothesize that social interaction in a group setting might be beneficial for obtaining and consolidating of cognitive skills. Furthermore, there is still an open question how the obtained skills can be generalized into the habitual community setting. So far, longitudinal studies have failed to corroborate the constant gains of cognitive remediation techniques in the everyday life of patients. Thus, meta-analysis demonstrates a small to moderate effect of cognitive rehabilitation on cognitive outcomes at the followup assessment in individuals with a diagnosis of schizophrenia [79].

The recent study of Penades et al. [80] showed that cognitive remediation therapy in schizophrenia patients may have a positive effect on the brain plasticity. Specifically, the authors demonstrated that brain network activation significantly changed and even tended to normalize during application of strategy-learning based treatment. Besides, there has been an increase of fractional anisotropy in the corpus callosum meaning potential improvement of interhemispheric information transfer. These data positively correlated with the parameters of cognitive functioning and functional outcomes. 

 Cognitive remediation has started to be applied in ultrahigh risk individuals with the aim of reducing and preventing the progression of schizophrenia. Meanwhile, only two studies have shown the beneficial effect of remediation on cognition in UHR individuals, although within the framework of an integrated psychological intervention [81]. In a study using computerized CogPack [82] the high-risk group displayed considerable gains in improving of cognitive skills, specifically in verbal memory in contrast to the patients with full psychosis. Furthermore, there is evidence from a study by Bechdolf et al. [83], who showed a significantly lower rate of transition to psychosis in the individuals that have been involved in integrated psychosocial interventions, which include cognitive remediation strategies. 

 Thus, the variety of techniques of cognitive remediation, blended with social skills training seems to be of the greatest utility in cognitive improvement in patients with psychosis, being particularly beneficial for clinically stable patients with schizophrenia and schizoaffective disorder. CR is becoming a useful tool in preventing cognitive decline in UHR individuals and boosting their social functioning. However, the durability of the gained positive effect of CR on cognition and social functioning must still be addressed in further longitudinal studies. 

## 4. Future Challenges for Cognitive Remediation

A notion that cognitive deficits are nuclear and irremediable is being challenged by the evidence of cognitive improvement following medication use and cognitive remediation therapy [84]. Cognitive impairment can still be moderate in the early stages of the schizophrenia prodrome and this supports current efforts to intervene in the early course of the illness [85]. However, the multifaceted nature of cognitive dysfunction raises the possibility of selecting more specific treatment targets in the future, specifically in slowing down the decline in working memory and executive functioning, improving the processing speed before the onset of psychosis [86]. A widespread cognitive impairment should not be assumed in all individuals with schizophrenia, as cognitive heterogeneity is likely to be present at the onset as well as throughout the illness. Although cognitive remediation may not contribute to the immediate gains in cognitive functioning, it could facilitate the retention of the improvements following treatment during all stages of schizophrenia.

It continues to be an aim of detection of individuals who are at ultrahigh risk of psychotic illness to treat them with cognitive techniques in order to prevent the further development of psychotic illness. The recent EDIE study has been reported as not significantly reducing transition to psychosis or symptom-related distress but has reportedly reduced the severity of psychotic symptoms in young people at high risk [87]. It could be that the use of the cognitive remediation techniques described may produce different results. Furthermore, while we continue to search for a new generation of antipsychotic drugs which will positively affect cognition, it seems probable that neurocognitive testing will begin to have a key role in the assessment of individuals with first episode or late prodromal psychosis, as a measurable correlate of the gray matter loss in these phases of the illness. 

In conclusion, the use of cognitive remediation could be an important part of treatment of the ultrahigh risk group in order to assist with changing cognitive deficits. Thus, a set of interventions, most likely combined therapy which incorporates cognitive remediation and social skills training, would be beneficial in slowing the progression of cognitive decline in UHR individuals. Moreover, given the risk of negative impact of cognitive deficits and social functioning in the prepsychotic stage, it is important to optimize the therapy by offering it over an adequate time. Such a comprehensive approach could maintain functional gains and preserve the opportunity to achieve functional improvement during the early stages of schizophrenia.
